# Effect of Multilaminate Small Intestinal Submucosa as a Barrier Membrane on Bone Formation in a Rabbit Mandible Defect Model

**DOI:** 10.1155/2018/3270293

**Published:** 2018-06-19

**Authors:** Weiyi Wu, Bowen Li, Yuhua Liu, Xinzhi Wang, Lin Tang

**Affiliations:** Department of Prosthodontics, Peking University School and Hospital of Stomatology, National Engineering Laboratory for Digital and Material Technology of Stomatology, China

## Abstract

A barrier membrane (BM) is essential for guided bone regeneration (GBR) procedures. Absorbable BMs based on collagen have been widely applied clinically due to their excellent biocompatibility. The extracellular matrix (ECM) provides certain advantages that can compensate for the rapid degradation and insufficient mechanical strength of pure collagen membrane due to the porous scaffold structure. Recently, small intestinal submucosa (SIS), one of the most widely used ECM materials, has drawn much attention in bone tissue engineering. In this study, we adopted multilaminate SIS (mSIS) as a BM and evaluated its in vivo and in vitro properties. mSIS exhibited a multilaminate structure with a smooth upper surface and a significantly coarser bottom layer according to microscopic observation. Tensile strength was 13.10 ± 2.56 MPa. In in vivo experiments, we selected a rabbit mandibular defect model and subcutaneous implantation to compare osteogenesis and biodegradation properties with one of the most commonly used commercial collagen membranes. mSIS was retained for up to 3 months and demonstrated longer biodegradation time than commercial collagen membrane. Quantification of bone regeneration revealed significant differences in each group. Micro-computed tomography (micro-CT) revealed that the quantity and maturity of bones in the mSIS group were significantly higher than those in the blank control group (*P* < 0.05) and were similar to those in a commercial collagen membrane group (*P* > 0.05) at 4 and 12 weeks after surgery. Hematoxylin and eosin staining revealed large amounts of mature lamellar bone at 12 weeks in mSIS and commercial collagen membrane groups. Therefore, we conclude that mSIS has potential as a future biocompatible BM in GBR procedures.

## 1. Introduction

Guided bone regeneration (GBR) is often used to achieve bone augmentation in preoperative alveolar bone loss. A barrier membrane (BM), which can prevent unwanted apical migration of soft tissue, is of great importance in the GBR procedure. Nonabsorbable membranes represented by polytetrafluoroethylene (PTFE) and titanium mesh have demonstrated good structural integrity over the course of the healing period [1]; however, the need for additional surgery to remove the membrane has been regarded as a disadvantage. In contrast, absorbable membranes that do not require secondary surgery for removal are widely used clinically, including synthetic polymer membranes, collagen membranes, and other natural polymer membranes, in which pure collagen membranes have attracted more attention in clinical use due to their excellent biocompatibility. However, previous studies have yet to reach a consensus on the mechanical and biodegradation properties of these membranes [2, 3]. Insufficient mechanical strength and an uncontrolled degradation period may lead to the collapse of the BM, as a result of invasion of epithelial tissue and interruption of new bone formation.

The extracellular matrix (ECM) is a complex scaffold consisting of not only collagens but also proteoglycans, glycoproteins, and glycosaminoglycans. The ECM demonstrates great structural integrity and is suitable for building porous scaffolds in tissue engineering due to excellent biocompatibility and biodegradability. Experimental studies have suggested that ECM material may enhance the regenerative capacity of the host tissue, specifically by interacting with the cell surface via numerous receptors and by mediating intracellular signaling pathways [4]. Small intestinal submucosa (SIS), a collagen-based membrane derived from ECM, provides a favorable environment for vascular endothelial cells to attach and proliferate [5]. For the past decade, SIS has been applied in tissue engineering for various tissues and organs, including the urinary bladder [6], the abdominal wall [7], tendons [8], and blood vessels [9]. The promotion of bone regeneration was first reported by Suckow in 1999 [10], indicating that fresh granular SIS provided the possibility of treating bone defects. The potential of SIS as an osteoconductive scaffold has attracted much attention during the past decade. In recent years, many studies have focused on the osteogenesis property of SIS, combining it with bone marrow stem cells (BMSCs) or adipose-derived stem cells (ADSCs) as a cell carrier to promote bone regeneration [11–13]. However, few studies have been conducted on SIS alone as a BM. In the repair of large-size defects, monolayer BMs tend to collapse into the defect area and affect the regeneration process [2]. Therefore, a multilaminate SIS (mSIS) obtained by stacking monolayer SIS in a specific way was adopted in this study, which can increase the mechanical strength while making the structure more three-dimensional. At present, there is still little research on whether mSIS can satisfy the requirements as a BM in GBR. For this reason, we hypothesized that a mSIS could be applied in GBR to prevent soft tissue invasion for a period of 3 months. The microarchitecture and mechanical properties were evaluated in vitro. Degradation properties and in vivo osteogenesis efficacy of the mSIS were also investigated in a rabbit mandibular model.

## 2. Materials and Methods

### 2.1. Morphology

To visualize from a histological viewpoint, mSIS specimens (Datsing Biological Technology Co., Ltd., Beijing, China) were embedded in paraffin and sectioned into 5-*μ*m thick slices using a sliding microtome (Microm HM200; Microm, Walldorf, Germany). Then mSIS was stained with hematoxylin and eosin (H&E) and examined using a light microscope (DP-72; OLYMPUS, Tokyo, Japan). To evaluate the surface morphology, mSIS and one of the most widely used commercial natural collagen membranes (Bio-Gide®; Geistlich Pharmaceutical, Wolhusen, Switzerland, hereafter called BG) were cut into pieces (5.0 mm × 5.0 mm) and observed using a field-emission scanning electron microscope (FESEM) (Supra55, Zeiss, Oberkochen, Germany) after sputter-coating with gold.

### 2.2. Mechanical Tests

mSIS and BG specimens were prepared in strips. Mechanical tests were performed on a universal testing machine (Universal Test Machine; Mecmesin Co., Slinfold, UK) at a crosshead speed of 10.0 mm/min. All tests were conducted at room temperature and repeated five times.

### 2.3. Biodegradation Properties

The experimental protocol was approved by the Animal Care and Use Committee of Peking University (Approval Number: LA2016264). To evaluate the biodegradation properties of mSIS, nine New Zealand rabbits (Center of Experimental Animal, Peking University School and Hospital of Stomatology), with a mean weight of 2.5–3.0 kg, were divided into three groups (n = 3) according to the observation interval. After general anesthesia (2% sodium pentobarbital administered at 30 mg/kg via intravenous injection) and localized disinfection, eight unconnected subcutaneous pouches were made on the back. Four sites were implanted with mSIS and another four with BG, in which three mSIS and three BG specimens were weighed before implantation, marked as *W*_1_. At 4, 8, and 12 weeks postoperatively, animals (n = 3 at each observation interval) were sacrificed. All residual specimens were collected. One mSIS and one BG specimen of each animal were immersed in 10% neutral formaldehyde solution and then gradually dehydrated in a series of ethanol solutions. After embedding the specimens in paraffin, they were sectioned into 5-*μ*m thick slices. The samples were stained with H&E and observed under a light microscope. The remaining three mSIS and BG specimens were preserved in 95% ethanol for 24 h, followed by washing three times with pure water. After drying in an air-dry oven until the weight was constant, specimens were weighed and marked as *W*_2_. The loss rate (marked as* w*) of mSIS or BG quality was calculated using the following formula:

(1) $$\begin{array}{c} \text{w}=\frac{W_{1}-W_{2}}{W_{1}}\times 100\% \end{array}$$

### 2.4. Rabbit Mandible Defect Model

Sixteen New Zealand rabbits were used for in vivo osteogenesis experiments. After general anesthesia using the same method above followed by depilation and disinfection, a parallel incision (about 3.0 cm in length) was made on the border of each side of the mandible. Separating subcutaneous tissues and a masseter muscle, a bone defect of 8 mm in diameter and 2 mm in depth was created using a turbo-drill on the body of the mandible, followed by cooling with physiological saline. Fragments of bone were washed to avoid potential bone self-regeneration. Bone defects (32 defects in total) were randomly divided into four groups: (1) BC group: it is blank control group, in which defects were left untreated; (2) BO group: defects were filled with deproteinized bovine bone mineral (Bio-Oss®; Geistlich Pharmaceutical, Wolhusen, Switzerland); (3) BS group: defects were filled with deproteinized bovine bone mineral and covered by mSIS; and (4) BG group: defects were filled with deproteinized bovine bone mineral and covered by BG. The incisions were sutured after implantation.

### 2.5. Gross Observation

At 4 and 12 weeks postoperatively, animals (n = 8 at each observation interval) were sacrificed, and bilateral mandibles were collected. The status of bone defect was observed for new bone formation and inflammation. Then, the mandible samples were preserved in a 10% neutral formaldehyde solution for further examination

### 2.6. Micro-Computed Tomography (Micro-CT) Evaluation

To measure new bone formation, the collected mandibles were scanned using a micro-CT scanner (SkyScan 1076; Bruker, Kontich, Belgium). The settings of the scanner were as follows: images were acquired at 70 kV, 149 *μ*A, with a pixel size of 18 *μ*m. The beam was filtered through a 0.5-mm aluminum filter. The scanned data were reconstructed, and three-dimensional images were obtained. The parameters of bone volume fraction (BV/TV, %) and bone mineral density (BMD, mgHA/mm^3^) were calculated for data analysis. Additionally, quantitative analyses of trabecular characteristics of different groups at 4 and 12 weeks after surgery, bone trabecular thickness (Tb.Th), trabecular number (Tb.N), and trabecular spacing (Tb.Sp) were also conducted using Inveon Research Workplace.

### 2.7. Histology

After micro-CT examination, all bone specimens were immersed in 10% neutral ethylenediaminetetraacetic acid (EDTA) solution for decalcification and then gradually dehydrated in a series of ethanol solutions. Specimens were embedded in paraffin and sectioned into 5-*μ*m thick slices using a sliding microtome. Then, samples were stained with H&E and Masson's trichrome. New bone formation and inflammation to host tissue at the defect site were evaluated using a light microscope.

### 2.8. Statistics

Statistical analyses were performed using IBM SPSS Statistics software (ver. 23.0; IBM Corp., Armonk, NY, USA). All quantitative data are expressed as means ± standard deviation (SD). Independent-samples t-test and one-way analysis of variance (ANOVA) were performed. A* P *value lower than 0.05 was considered statistically significant.

## 3. Results

### 3.1. Morphology

**Figure 1:**
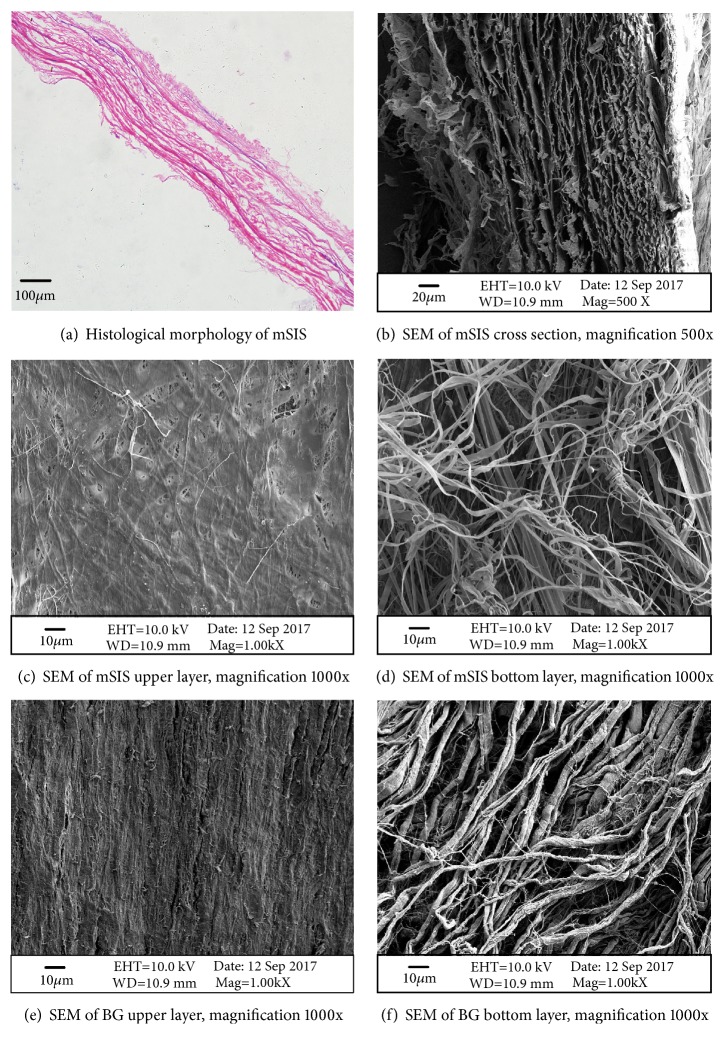
Histological morphology and SEM images of mSIS and BG.

In the histological analysis of mSIS, a large number of collagen fibers were detected with no cells. Cross-sectional images indicated a multilayered structure for mSIS [Figure 1(a)]. Scanning electron microscopy (SEM) images also showed the mSIS to be multilayered [Figure 1(b)]. One side of the mSIS seemed smoother and denser [Figure 1(c)], whereas the opposite face exhibited a grid-like interconnected structure with multiple pores [Figure 1(d)], significantly coarser than the smooth side. BG also showed different structures on two sides: a smooth upper surface [Figure 1(e)] and a rougher bottom layer with collagen strands [Figure 1(f)].

### 3.2. Mechanical Strength

The average tensile strength of mSIS was 13.10 ± 2.56 MPa, whereas that of BG was 7.23 ± 2.05 MPa. The tensile strength of mSIS was significantly higher than that of BG (*P* < 0.05).

### 3.3. Biodegradation Properties

**Figure 2:**
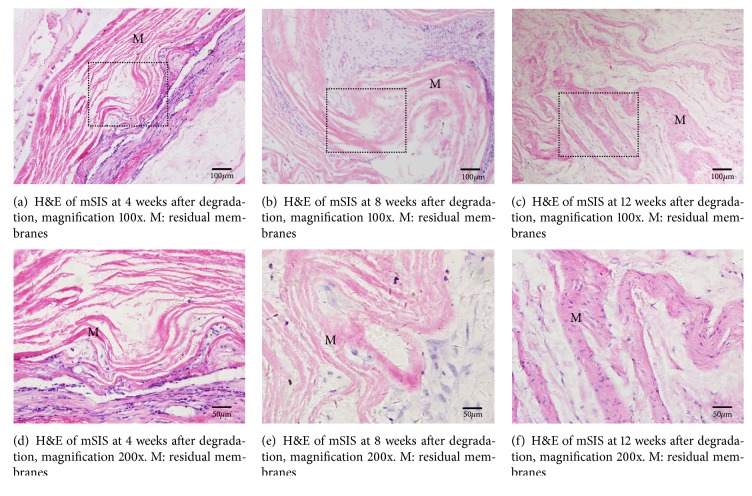
Hematoxylin and eosin (H&E) staining of mSIS at different times after implantation.

**Figure 3:**
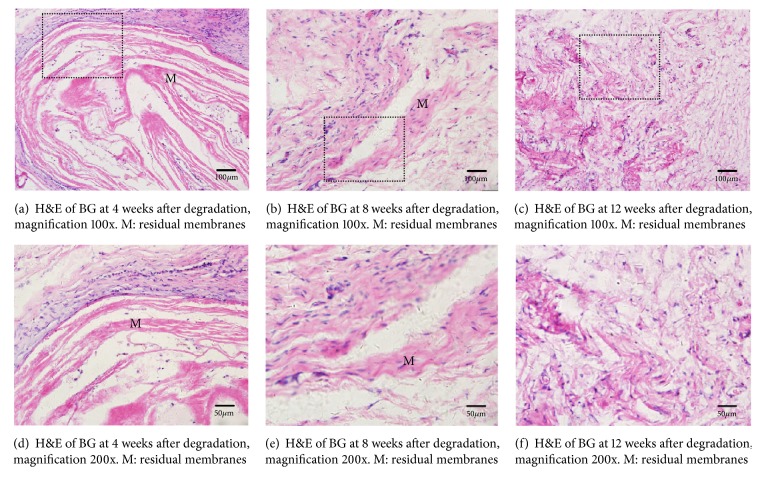
H&E staining of BG at different times after implantation.

**Figure 4:**
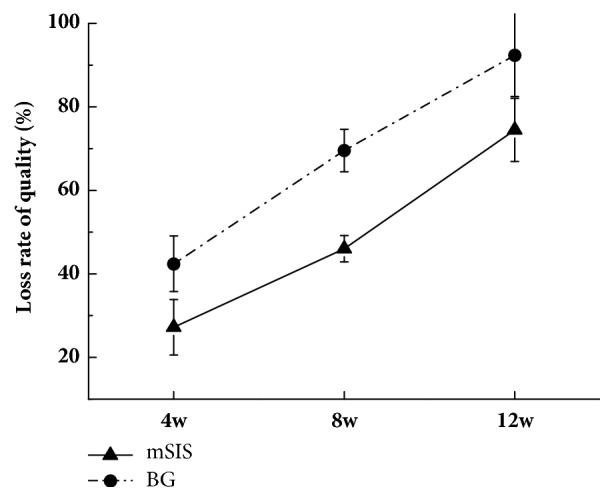
Loss rate of quality for mSIS and BG at 4, 8, and 12 weeks after implantation.

In vivo subcutaneous implantation experiments revealed no sign of postoperative infection or membrane exposure. No specific changes in mSIS or BG were visible to the eye, and specimens of mSIS and BG seemed intact and easy to peel 4 weeks after implantation. mSIS stayed intact but partly adhered to the surrounding tissue 8 weeks postoperatively, whereas BG was broken with only discontinuous debris remaining. At 12 weeks after implantation, mSIS was incomplete and difficult to separate from surrounding tissue, whereas BG was almost invisible. Figures 2 and 3 show histological staining of mSIS and BG at different times after implantation. At 4 weeks after surgery, microscopic observation revealed structural integrity with a clear margin to the surrounding tissue of mSIS and BG [Figures 2(a) and 3(a)], with a few inflammatory cells infiltrated around the membranes [Figures 2(d) and 3(d)]. At 8 weeks after operation, collagen fibers of mSIS were still intact and were partly integrated with connective tissue [Figure 2(b)], whereas BG was broken and only a few residual fibers could be found under the microscope [Figure 3(b)], in accordance with gross observation. No obvious inflammatory infiltration was observed in mSIS or BG groups at this time [Figures 2(e) and 3(e)]. At 12 weeks after implantation, H&E staining revealed that collagen fibers of mSIS were nearly integrated with surrounding tissue [Figure 2(c)], whereas BG could hardly be found microscopically [Figure 3(c)].  Figure 4 shows the rates of loss of quality for mSIS and BG.

### 3.4. Gross Observation

**Figure 5:**
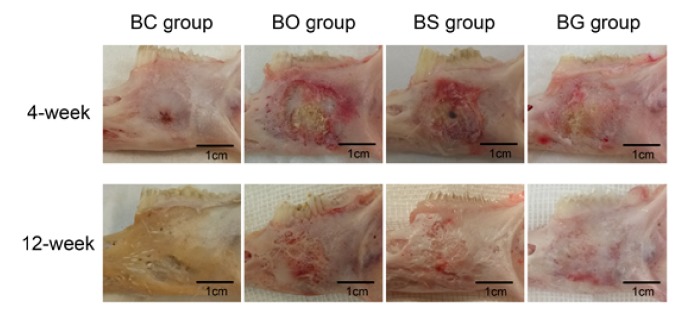
Gross observation of specimens at 4 and 12 weeks after surgery.

Figure 5 shows the gross view of specimens at 4 and 12 weeks postoperatively. In general, no sign of postoperative infection or membrane exposure was observed. The BC group still remained with an unhealed cavity at 4 and 12 weeks after surgery, indicating that the defect model we created was a “critical-size defect.” New bone formation was evident in the BO, BS, and BG groups at 4 weeks after surgery, with the defect margin still clearly visible. Twelve weeks after surgery, the defect in the BO group was almost healed, with a rather rough surface and indistinct margin. The defect area was integrated with autogenous bone in the BS and BG groups at 12 weeks postoperatively; the margin was invisible, leaving a flat smooth surface.

### 3.5. Micro-CT Evaluation

**Figure 6:**
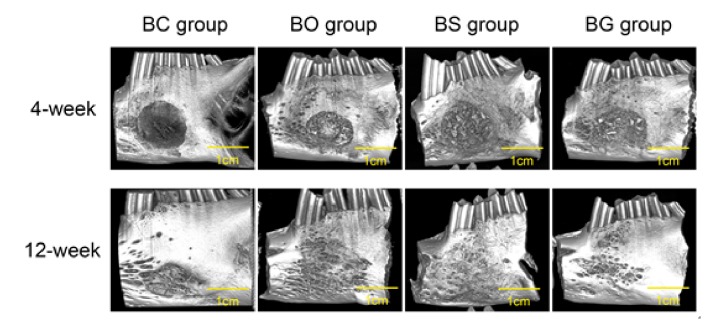
Three-dimensional micro-computed tomography (micro-CT) images of specimens at 4 and 12 weeks after surgery.

**Figure 7:**
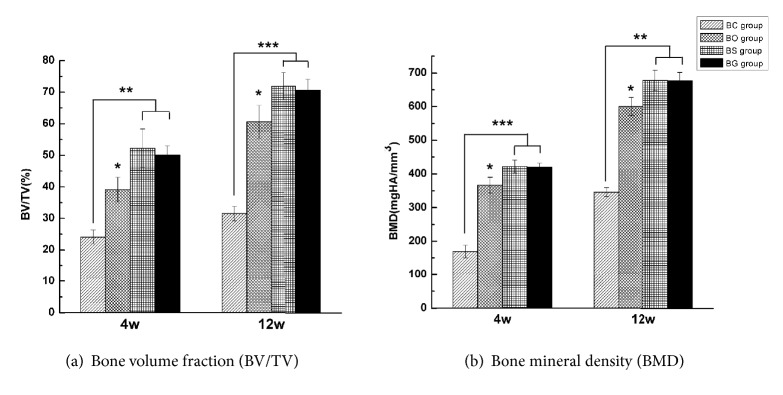
Quantification graph of specimens at 4 and 12 weeks after surgery. ^*∗*^*P* < 0.05, ^*∗∗*^*P* < 0.01, and ^*∗∗∗*^*P* < 0.001.

Figure 6 shows three-dimensional images of specimens at 4 and 12 weeks after surgery, which were in accordance with gross observation. At 4 weeks after surgery, the defect area of the BC group was almost empty, whereas new bone formation could clearly be seen in the BO group, as well as the BS and BG groups. Additionally, high-density residual particles were left within the margin of the defect, indicating that the deproteinized bovine bone mineral did not degrade completely at 4 weeks. BS and BG groups at 12 weeks after surgery exhibited the best healing state, with a flatter surface and invisible defect margin. The defect area in the BO group at 12 weeks was basically healed, and the surface was rougher than that of the BS or BG group. Quantification of bone regeneration revealed significant differences in each group (Figure 7). The BV/TV and BMD were significantly higher in BS and BG groups than in BC and BO groups at 4 and 12 weeks after surgery (*P* < 0.05).

Table 1 lists micro-CT bone morphometry data at 4 and 12 weeks after surgery. The greater the value of Tb.Th and Tb.N, the more mature and stable the bone structure. In contrast, Tb.Sp stands for the degree of connectivity of trabecular bone; if the value of Th.Sp is bigger, the arrangement of the bone structure would be worse. As shown in Table 1, at postoperative weeks 4 and 12, the Tb.Th and Tb.N of newly formed trabecular bone in BS and BG groups were significantly higher than those in the BC and BO groups (*P* < 0.05). In contrast, the values of Tb.Sp in BS and BG groups were significantly lower than those of BC and BO groups, indicating that mature and well-connected bone tissue had formed in the BS and BG groups at 12 weeks after surgery (*P* < 0.05).

### 3.6. Histology

**Figure 8:**
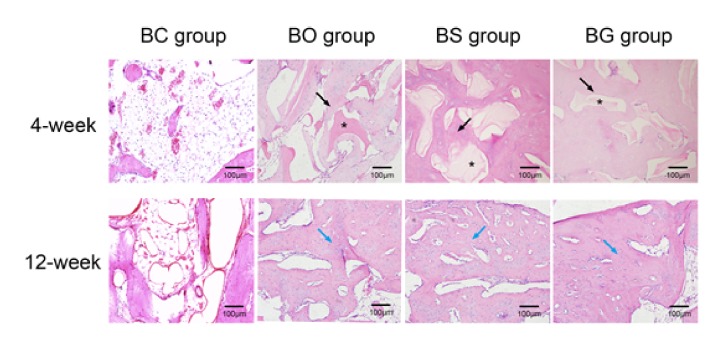
H&E staining of the defect site at 4 and 12 weeks after surgery. Black arrow: newly formed bone; blue arrow: mature lamellar bone; asterisk: residual particles of the deproteinized bovine bone mineral.

**Figure 9:**
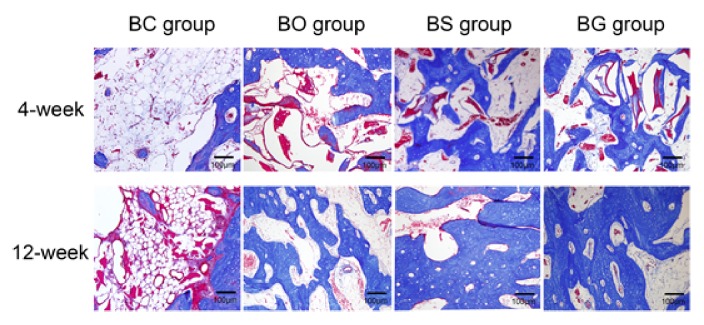
Masson's trichrome staining of the defect site at 4 and 12 weeks after surgery.

Figure 8 shows H&E staining of the defect site. At 4 weeks after surgery, a large number of lipid vesicles were found in the BC group, with a small quantity of inflammatory cells infiltrating the defect area, whereas a small amount of newly formed bone was seen at the edge of the defect area. In the BO group, residual particles of the deproteinized bovine bone mineral were observed (Figure 8, asterisk), with immature bone formation (Figure 8, black arrow). More new bone formation was found in the BS and BG groups; granules of deproteinized bovine bone mineral remained as well. At 12 weeks after the operation, fibrous tissue, lipid vesicles, and only a small amount of mature bone were found in the defect site in the BC group. A large amount of mature lamellar bone (Figure 8, blue arrow) was observed in tBS and BG groups, with bone lacunae formation. Mature new bone formation could also be found in the BO group; however, the area of new stained bone was smaller than that in BS and BG groups. Masson's trichrome staining mainly stains collagen fibers, as shown in Figure 9, which was in accordance with H&E staining. Large amounts of lipid vesicles and connective tissue were observed at 4 and 12 weeks postoperatively. A small number of woven bones and some residual materials were observed in the BO group at 4 weeks, and newly formed bone was much more prominent at 12 weeks after surgery. In the BS and BG groups, immature bone islands were observed at 4 weeks, with large amounts of lamellar bone formed at 12 weeks.

## 4. Discussion

An ideal BM should be biocompatible, biodegradable, osteoconductive, osteoinductive, convenient to use, and affordable [14]. There are two key reasons for the success of GBR technology: prevention of epithelial tissue from apical migration and maintenance of the defect space. A BM should be two-sided, with one side sufficiently dense to prevent soft tissue from invading the defect site and the other with a porous structure for better infiltration and cell adhesion [15]. In this experiment, the mSIS membrane used consisted of eight layers of acellular porcine SIS, in the form of a lyophilized stack. With regard to the manufacturing process of the mSIS, porcine small intestine was obtained and harvested from healthy home-raised pigs within 4 h of sacrifice. Intestine processing involved mechanical disassociation, degreasing, enzyme digestion, detergent treatment, and lyophilization to remove the mucosa, myometrium, and serosal layer, together with adipose tissue and cellular components, leaving the basic structure of the submucosa [16]. After that, each layer of SIS was stacked in a specific way to obtain a mSIS. All samples were freeze-dried and then sterilized under ethylene oxide. Cytotoxicity evaluation was performed using MTT assay; mSIS revealed only a slight cytotoxicity and was safe to use in vivo. In this study, SEM images showed the mSIS to be smooth and dense on one side, whereas the opposite side had a grid-like interconnected structure with multiple pores, which was in accordance with previous studies [17]. Cross-sectional images and histological staining revealed that the mSIS layers were closely integrated.

We attempted to remove all cellular components of the mSIS used in this experiment to reduce immunogenicity while retaining the intact ECM framework to support host cells, because the presence of xenogeneic ECM in SIS will lead to infiltration of mononuclear cells, thus initiating an inflammatory response [18]. As we can see from H&E staining of mSIS, no cells could be found. In the in vivo experiment, soft tissue contact with the mSIS membrane at 4 weeks led to a transient inflammatory reaction and local inflammatory cell infiltration. At 12 weeks after implantation, the mSIS membrane was basically integrated with the surrounding soft tissue without obvious inflammatory cells and exhibited good biocompatibility.

In this study, we found that the tensile strength of multilaminate SIS under wet conditions was significantly higher than that of BG, showing better mechanical properties compared with the commercial collagen membrane. Studies have shown that the mechanical strength of collagen membrane, such as BG, degrades after wetting and has the potential to collapse into the defect area 4 to 6 weeks after GBR surgery [2]. Notably, the success of the GBR procedure depends on space maintenance for a sufficient period (of at least 6 weeks) to allow for new bone formation, depending on the size of the defect [19, 20]. Premature degradation of the BM or insufficient mechanical strength will cause soft tissue invasion. Some collagen membrane techniques incorporate a cross-linking agent during production to improve mechanical and degradation properties [21]. This method, however, has the possibility of leading to cytotoxicity and calcification if the cross-linking agent remains [22, 23]. Previous studies have shown that cross-linked absorbable membranes may affect the process of vascularization during bone formation [24]. In addition to type I and type III collagen, SIS also has a three-dimensional helix of collagen fibers and elastic fibers so as to resist the stress of the surrounding tissue [25]. This membrane also maintains a certain degree of tension after wetting and is less prone to collapse or displacement. Castilla et al. noted that the ideal porosity of a bone tissue engineering scaffold should be higher than 80% to promote vascularization and bone regeneration [26]. However, the increase in porosity inevitably degrades the mechanical properties. Therefore, establishing a multilaminate scaffold can help to achieve excellent mechanical properties as well as suitable porosity, while increasing the osteoconductivity.

The degradation properties of an eight-layer multilaminate SIS were examined subcutaneously in this study. Mewaldt et al. conducted in vitro degradation experiments of SIS of different layers using collagenase-containing aqueous solution to simulate the wound environment [27]. The results showed that the degradation rate of SIS was inversely proportional to the number of layers. However, few studies have systematically focused on the degradation time of multilaminate SIS. After 8 weeks of implantation, the morphology of mSIS remained intact, and the loss rate of quality was less than 50%. Twelve weeks after implantation, the degradation rate of mSIS was 74%; this was basically consistent with the results of Gilbert et al., who adopted ^14^C labelling to study the degradation of SIS indirectly [28]. As for the commercial collagen membrane, BG degraded 65% after 8-week implantation; severe degradation led to the complete absence of the membrane after 12 weeks. Previous studies also investigated the biodegradation properties of BG. Histological staining revealed light-to-moderate degradation after 1 month and moderate-to-severe degradation 2 months after implantation [29], which was in agreement with our study. Therefore, we confirmed that multilaminate SIS can effectively prolong the degradation time compared with BG, the commonly used commercial collagen membrane, which meets the requirement of degradation properties of GBR.

We adopted multilaminate SIS as a BM in a rabbit critical mandible defect model, confirming that mSIS could effectively promote bone regeneration as a BM in the defect area. As is well known, bone healing and regeneration is a complex process, consisting of a sequence of biological events, including inflammation, cell recruitment, and bone formation and remodeling [30]. Turri et al. discovered the frequent appearance of giant multinucleated and osteoclast-like cells, particularly in the zone between the lower surface of the membrane and the bottom of defect area when adopting SIS to repair a rat femur defect [31]. Cell migration and cytokine release were promoted by SIS in in vitro studies [32, 33], suggesting that the SIS membrane acts as a bioactive compartment rather than merely a passive barrier.

At 4 weeks after the operation, bone defects of the blank control group maintained the cavities with little change in diameter. After 12 weeks, the defect area had shrunk but was still visible, confirming that the defect model we created met the requirement of “critical-size defect”. In this study, we adopted BMs combined with the Bio-Oss granules to improve bone regeneration, simulating the GBR procedure clinically. Bio-Oss is the trade name of deproteinized bovine bone mineral, which provides an osteoconductive scaffold and supports the barrier membrane from collapsing into the defect area, which has been confirmed by previous studies [34–36]. In the healing of a bone defect, distance osteogenesis is defined as new bone formation from an existing bone, such as the healing of a fresh extraction socket, while contact osteogenesis is the formation of bone on the surface of the implant, bone substitute material, or BM that increases bone formation of the area due to osteoconductivity [37, 38]. Histological staining in this study indicated new bone formation around the bone substitute material and beneath the membrane, while only a small amount of new bone at the edge of the defect could be found in the blank control group. Additionally, micro-CT results suggested that the values of BV/TV and BMD of the membrane-covered group were significantly higher than those of the BO group, mainly due to the presence of the BM to prevent the loss of deproteinized bovine bone mineral, which was supported by previous studies [39].

Previous studies have shown that bone microstructure plays a crucial role in the quality of bone after healing. In 2010, Bouxsein et al. reported that Tb.Th, Tb.Sp, and Tb.N were the most crucial parameters of indicating the microstructure of trabecular bone [40]. Hsu et al. reported similar results: the thickness and separation of trabecular bone were the most crucial parameters to evaluate the microarchitecture of new bone [41]. These parameters played an important role in the stability and long-term success of bone healing. Similar results were reported in our study of trabecular bone. BS and BG groups at 12 weeks had a larger quantity and more highly three-dimensional network of trabecular bone, which was regularly and closely arranged.

This study provides a preliminary experimental basis for the application of mSIS as a barrier membrane in GBR technology. However, it can be seen from the results that the standard deviation of mSIS group was slightly higher than that of BG group, indicating that the consistency of SIS donors as well as the preparation process still needs to be improved. In addition, further researches are still necessary to verify the osteogenic properties of mSIS.

## 5. Conclusion

In conclusion, we demonstrated that multilaminate SIS possesses a porous microstructure and excellent mechanical and biodegradation properties and can promote bone regeneration, revealing its potential for bone repair and promising prospect in clinical guided bone regeneration technology. However, it is still necessary to perform more studies to explore the mechanism of bone regeneration before further clinical applications of mSIS.

## Figures and Tables

**Table 1:** Micro-CT quantitative analysis of trabecular characteristics of different groups at 4 and 12 weeks after surgery (mean ± standard deviation).

		BC group	BO group	BS group	BG group
Tb.Th	4w	0.069 ± 0.017	0.123 ± 0.024^*∗*^	0.151 ± 0.015^*∗*^	0.137 ± 0.017^*∗*^
	12w	0.142 ± 0.037	0.226 ± 0.018^*∗*^	0.292 ± 0.045^*∗*△^	0.288 ± 0.019^*∗*△^
Tb.N	4w	0.515 ± 0.073	0.732 ± 0.069^*∗*^	0.925 ± 0.089^*∗*△^	0.909 ± 0.078^*∗*△^
	12w	1.188 ± 0.213	1.545 ± 0.056^*∗*^	1.886 ± 0.089^*∗*△^	1.850 ± 0.097^*∗*△^
Tb.Sp	4w	1.074 ± 0.170	0.818 ± 0.084^*∗*^	0.567 ± 0.061^*∗*△^	0.584 ± 0.028^*∗*△^
	12w	0.758 ± 0.115	0.389 ± 0.040^*∗*^	0.215 ± 0.055^*∗*△^	0.234 ± 0.049^*∗*△^

Tb.Th and Th.Sp: units of mm; Tb.N: unit of mm^−1^.

^*∗*^Compared with the BC group,* P *< 0.05.

^△^Compared with the BO group, *P *< 0.05.
